# Adolescence internalizing problems as a mediator between autism diagnosis in childhood and quality of life in emerging adults with and without autism: a 10-year longitudinal study

**DOI:** 10.1186/s12888-023-04635-w

**Published:** 2023-03-09

**Authors:** Per Normann Andersen, Stian Orm, Ingrid Nesdal Fossum, Merete Glenne Øie, Erik Winther Skogli

**Affiliations:** 1 Department of Psychology, Inland Norway University of Applied Sciences, Holsetgata, Norway; 2 Division Mental Health Care, Innlandet Hospital Trust, Brumunddal, Norway; 3 Department of Psychology, University of Oslo, Oslo, Norway; 4 Research Department, Innlandet Hospital Trust, Brumunddal, Norway

**Keywords:** Autism, Quality of life, Internalizing, Externalizing, longitudinal

## Abstract

**Background:**

Individuals with autism tend to have a reduced quality of life across their lifespan. This reduced quality of life may be a result of autism traits, mental distress, and poor person/environment fit. In this longitudinal study, we looked at the role of adolescence internalizing and externalizing problems in mediating the relationship between having an autism diagnosis from childhood and perceived quality of life as emerging adults.

**Methods:**

A total of 66 participants in a group of emerging adults with autism (mean age 22.2 years), and without autism (mean age 20.9 years) were assessed in three assessment waves (T1 at 12 years, T2 at 14 years, and T3 at 22 years of age). Parents filled out the Child Behavior Checklist at T2 and participants filled out the Perceived Quality of Life Questionnaire at T3. Total and indirect effects were tested in serial mediation analysis.

**Results:**

The results showed that internalizing problems fully mediated the association between childhood autism diagnosis and the quality of life in emerging adulthood, while externalizing problems did not.

**Conclusion:**

Our findings suggest that attention to adolescent internalizing problems is important to improve the later quality of life for emerging adults with autism.

## Background

Autism is considered a lifelong condition related to multiple origins, with both genetic and environmental factors playing an important role [1]. Having an autism diagnosis is commonly perceived as a stigma and associated with poorer psychological well-being from childhood through adolescence [2, 3]. Problems related to adaptive functioning, social integration, and independent living are common for people with autism 1[4, 5]. Further, an autism diagnosis is considered to qualify for a significant burden of daily life and reduced quality of life (QoL) and health problems across the lifespan for individuals with autism [6]. In particular, the period of emerging adulthood gives rise to poor outcomes related to both subjective and objectively measured QoL for many people with autism [7, 8].

This period of emerging adulthood is characterized by fundamental changes within personal, social, emotional, neuroanatomical, and developmental levels. Emerging adulthood is the period of life between adolescence and young adulthood (i.e., late teens – mid to late 20s), typically characterized by identity exploration, instability, self-focus, and a feeling of being in-between adolescence and being an adult [9]. Parallel with the described changes and expectations of increased independence, familial and institutional support is gradually withdrawn. As a result of this, emerging adulthood can be considered a critical phase in human development with a significant impact on QoL [10], QoL refers to an individual’s perception of the quality of their health, relationships, school/job satisfaction, and participation in society [11]. It is a subjective assessment of well-being and how one feels about everyday functioning concerning physical, cognitive, and social domains [12].

Children, adolescents, and adults with autism report poorer subjective QoL throughout their lifespan compared to neurotypical individuals [13–17]. Important factors for the low QoL in autism are high levels of stress, depressive symptoms, a history of being bullied, low levels of independence in everyday life, lack of support for everyday challenges related to autism symptoms, sleep problems, impaired executive functioning, female sex, and experiencing stigma related to diagnosis [18–22]. Although there are many causes of poor quality of life, an autism diagnosis has been linked to poor quality of life in adolescence and emerging adulthood [23]. Furthermore, there is evidence that emerging adults with autism rate their QoL as lower than emerging adults with other psychiatric disorders, such as attention-deficit/hyperactivity disorder, disruptive behavior disorders, and affective disorders [8]. Adults with autism seem to have lower rates of independent living, lower educational levels, less paid employment, and a higher reliance on social welfare than emerging adults with a psychiatric disorder without autism. These findings may indicate a significant negative effect of autism on QoL [8]. However, in contrast to this, Chiang and Wineman [24] found in their review of QoL in autism that a majority of studies reported good QoL in adults with autism without intellectual disability and that autism was not related to QoL. A recent study also indicates large individual variability concerning QoL in emerging adults with autism without intellectual disability [19]. While the findings are inconsistent, some studies have suggested that higher age is associated with lower QoL in individuals with autism [14, 25]. Therefore, it is important to investigate QoL within specific developmental periods, such as emerging adulthood. Previous studies have also had the limitation of investigating samples with a wide age range, spanning several developmental stages of adulthood (see [26] for a review). Furthermore, lower levels of QoL among individuals with autism emphasize the importance of identifying factors associated with this experience.

One possible factor contributing to reduced QoL in autism is the stigma related to having autism, which has been found to be a significant contributor to the mental health of people with autism [3]. In a qualitative study by Botha, Dibb [22], adult participants with autism reported that they experienced not having a proper childhood because of trying to alleviate the effects of stigma associated with receiving an autism diagnosis. They reported feeling like failures as a result of their desire and relentless attempts to assimilate. Furthermore, we know that having experienced mental health difficulties, such as autism, in childhood, is related to lower well-being in emerging adulthood [12].

Moreover, co-occurring psychopathology symptoms have been found to predict poorer QoL for people with autism [14, 19, 27–29]. Two commonly used dimensions of psychopathology are internalizing (e.g., anxiety and depressive symptoms) and externalizing (e.g., rule-breaking behavior and conduct disorders) problems [30]. Internalizing problems such as anxiety and depression seem to increase the negative effect of autism on QoL in adolescents with autism [19]. From a conceptual, clinical, and empirical point of view, internalizing problems may mediate the relationship between autism and externalizing problems. Clinical observations and conceptual models of co-occurring psychopathology symptoms in individuals with autism have suggested that aspects of externalizing problems, such as aggressive behavior and acting out, could be expressions of negative affectivity due to anxiety and/or depression [31]. This point of view is supported by empirical findings showing that internalizing problems mediated the relationship between autism and externalizing problems in adolescence [32], and that symptoms of anxiety and depression, such as worrying and rumination, predict later externalizing behavior in youth with autism [33]. Thus, it could be that internalizing problems have an externalizing expression in individuals with autism[31, 34] and, consequently, it could be that internalizing problems mediate the relationship between autism and externalizing problems. Externalizing problems may still have an independent contribution to well-being and QoL among individuals with autism. For example, externalizing problems have been found to contribute beyond internalizing problems to the prediction of suicidal behaviors in youth with autism [34]. Studies reporting on the association between externalizing problems and QoL in people with autism without intellectual disabilities are scarce, but there is some evidence that externalizing problems negatively affect QoL in autism, potentially by the capability of externalizing problems to disrupt social relationships [35]. Studies of potential mediators and moderators are especially valuable because, by identifying mediators and moderators, we can better develop and target interventions for improving QoL among individuals with autism.

## Current study

In this longitudinal study, we followed individuals with and without autism diagnosis (IQ > 70) in three assessment waves over 10 years from childhood into emerging adulthood. We aimed to investigate how adolescent internalizing and externalizing problems mediated the relationship between receiving an autism diagnosis in childhood and QoL in emerging adulthood, which to our knowledge has not been studied yet. First, due to mixed evidence, we made no clear hypothesis regarding the effect of autism diagnosis on QoL. Second, we hypothesized that internalizing problems mediate a possible effect of autism diagnosis on reduced QoL. Third, we hypothesized that externalizing problems mediate a possible effect of autism diagnosis on reduced QoL, independently of internalizing problems. Fourth, if hypotheses two and three are supported, we expect both internalizing and externalizing problems to act as mediators in succession where internalizing problems influence externalizing problems, as internalizing problems are more pronounced among emerging adults with autism diagnosis and have been found to mediate the relationship between autism and externalizing problems [32, 36]. However, we will reverse the ordering in a separate analysis as this succession is uncertain. Lastly, if either internalizing or externalizing problems are identified as significant mediators, the effect of subscales (somatic complaints, withdrawn-depressed, anxious-depressed) as parallel mediators will be explored.

## Method

### Participants and procedure

The participants in this study are part of the Lillehammer Neurodevelopmental Follow-Up Study (LINEUP), a 10-year longitudinal study on cognitive and emotional development in autism, Tourette’s Syndrome (TS), Attention-Deficit/Hyperactivity Disorder (ADHD) and a neurotypical comparison group (CG). In the current study, data from the autism and CG participants were of interest. The participants were recruited as children (mean age 12 years) from child and adolescent psychiatric outpatient clinics at Innlandet Hospital Trust in Norway in 2008–2009. Participants were excluded if born prematurely (< 36 weeks), had a disease affecting the central nervous system, or had IQ < 70. In total, 38 individuals with autism spectrum disorder who met the Diagnostic and Statistical Manual of Mental Disorders, fourth edition (DSM-IV) criteria were recruited, along with 50 neurotypical participants in the comparison group (CG) (for details regarding assessment see Andersen: [37]).

At two-year follow-up 87 participants (autism = 37, CG = 50) were available. Sixty-six of the participants (autism = 26, CG = 40) were available for follow-up after 10 years (total retention 76%). The mean ages at ten years follow-up were 22.2 years (SD 2.6) for the autism group and 20.9 years (SD 1.9) for the CG ( for details see [38]). The differences between groups were tested with independent samples T-tests, where Bonferroni corrected *p* values below 0.05 / 3 = 0.017 were considered statistically significant. The results indicated no statistically significant differences (see Table 1) between the two groups in terms of age (*p* = .024), Full -scale IQ (*p* = .114) or gender (*p* = .167). Differences between those available for follow-up after 10 years and those not retained were tested with independent samples T-tests, where Bonferroni corrected *p* values below 0.05 / 7 = 0.007 were considered statistically significant. The results indicated no statistically significant differences between the two groups in terms of age (*p* = .842) and FSIQ (*p* = .069) at baseline (T1) or internalizing symptoms (*p* = .343), externalizing symptoms (*p* = .533), anxious/depressed (*p* = .560), withdrawn/depressed (*p* = .195) and somatic complaints (*p* = .235) at the two-year follow-up (T2).

**Table 1:** Demographic characteristics at 10-year follow-up

	Autism (*n* = 26)		CG (*n* = 40)		*p*
	*M*	*SD*	*M*	*SD*	
Gender (male/female)	21/5		26/14		.167
Age	22.1	2.6	20.9	2.6	.024
FSIQ	109.3	18.3	115.4	12.0	.114

Note. Age in years. CG = neurotypical comparison group. FSIQ = Full-Scale IQ, estimated from Wechsler Abbreviated scale of Intelligence. Corrected p level due to multiple comparisons 0.5/3 = 0.017

The diagnostic assessment at baseline (T1) was based on separate interviews with children and parents, using the Schedule for Affective Disorders and Schizophrenia for School-Aged Children/Present and Lifetime version-2009 (K-SADS-PL; 39). K-SADS-PL is a semi-structured diagnostic interview based on the DSM-IV [40], consisting of a screening interview and eight diagnostic supplements. In addition, the parents completed the Autism Spectrum Screening Questionnaire (ASSQ; [41]). ASSQ has excellent test-retest reliability, inter-rater reliability, sensitivity, and specificity all ranging from 0.62 − 0.91 [41, 42]. This diagnostic procedure was repeated after two years (T2), see Andersen, Skogli [43] for further details regarding the diagnostic procedure.

The diagnostic assessment at 10-year follow-up (T3; [44]) was based on the Asperger Syndrome Diagnostic Interview (ASDI; [45]) and the Autism Spectrum Quotient 10-item (AQ 10; [46]). The clinical interview was conducted by experienced clinicians highly familiar with autism. Information from the interview was then discussed in a team with a clinical psychologist, educational therapist, and supervising clinical neuropsychologist specialized in neurodevelopmental disorders, to determine whether a participant still met the DSM-IV criteria for an autism diagnosis based on fulfilled criteria on the ASDI or the cut-off at the AQ-10. Four participants were not re-assessed with ASDI as they only wished to take part in the online questionnaire part of the follow-up, and assessment with ASDI required physical participation. Of those re-assessed, 19 out of 22 met autism cut-off criteria at either ASDI or AQ -10. In lack of Norwegian norms, we did a comparison of the AQ-10 scores between our autism group and a group of emerging adults from a Swedish general population sample [68]. The results revealed a significant and large difference in autism symptoms based on AQ-10, where our sample displayed more autism symptoms compared with Swedish general population adults (*t* (25) = 7.961, *p* < .001, *d* = 1.56).

### Measures

#### Quality of life

Quality of life (QoL) was measured at T3 using the Norwegian version of The Perceived Quality of Life Scale (PQoL; [12, 47, 48]). PQoL is a self-report questionnaire assessing the physical, social, and cognitive quality of life. It consists of 20 different questions using a Likert scale from 0 (extremely dissatisfied) to 10 (extremely satisfied). The total score (see Table 2) represents the sum of the physical, social, and cognitive subscales for each group of participants, with higher scores indicating better quality of life. The present version of PQoL was constructed to measure life satisfaction within areas of life considered important for people with varying degrees of wellness and disability [48]. The psychometric properties of the Norwegian version have been reported as good with a Cronbach’s alpha of 0.93 [47].

**Table 2:** T-scores on the Child Behavior Checklist and raw scores on Perceived Quality of Life

	Autism (*n = 26)*		CG (*n = 40)*	
	*M*	*SD*	*M*	*SD*
Internalizing problems^1^	59.8	10.9	41.1	8.3
Externalizing problems^1^	50.2	8.9	39.7	5.6
Somatic complaints^1^	56.7	8.1	52.6	5.0
Withdrawn/depressed^1^	63.7	7.8	50.7	1.6
Anxious/depressed^1^	59.5	9.9	51.1	2.5
Perceived quality of life^2^	118.5	26.8	134.0	17.2

Note. CG = comparison group. 1Higher scores indicate greater problems, Child Behavior Checklist (CBCL). 2Higher scores indicate better perceived quality of life, Perceived Quality of Life scale (PQoL).

#### Co-occurring internalizing and externalizing problems

Co-occurring internalizing and externalizing problems were measured at T2 using the Child Behavior Checklist (CBCL; [49]). The CBCL yields a total score of emotional and behavioral problems and comprises broader band scales of internalizing (subscales of anxiety/depression, withdrawn/depression and somatic complaints) and externalizing (subscales of rule-breaking and aggression) problems. Raw scores are converted to norm-referenced *T*-scores (*M* = 50, *SD* = 10). There are no national CBCL norms in Norway, but the scores from Norwegian children are lower compared to American children, and Norway is a so-called low-scoring country with a population mean well below a T-score of 50 [50]. Higher T-scores indicate a higher degree of problems. The CBCL has demonstrated good psychometric properties both internationally [51] and for the Norwegian version with good sensitivity (40–83%), specificity (70–94%) and internal consistency (Cronbach’s alpha ≥ 0.8) [50, 52].

#### Intellectual functioning

Estimated Intellectual functioning (estimated full-scale IQ) was measured using the Wechsler Abbreviated Scale of Intelligence (WASI; [69]).

### Data analysis

**Fig. 1:**
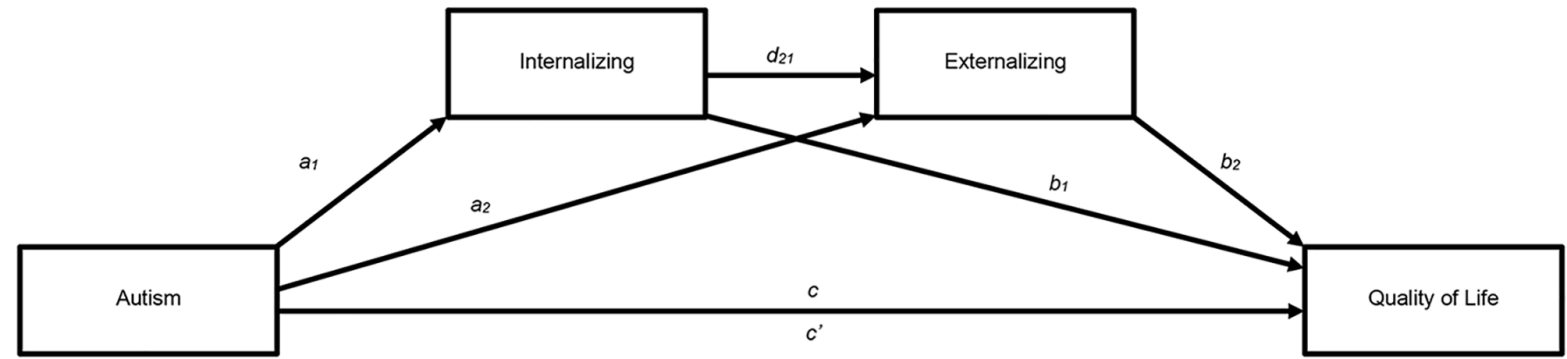
Conceptual serial mediation model where c represents the total effect (direct and indirect through mediators) between the predictor (autism) and the outcome variable (Quality of Life). Paths a_1_ and a_2_ represent the relationship between the predictor and mediators (internalizing and externalizing). Path d_21_ represents the relationships between the two mediators. b_1 and_ b_2_ represent the relationship between the mediators and outcome variables after controlling for the predictor. The c^’^ path is the direct effect between the predictor and the outcome variable without the effect of the mediator variables. The indirect effect (c - c^’^ = a_1_ b_1_ + a_2_ b_2_ + a_1_ d_21_b_2_) is indicated through a statistically significant (i.e. CIs does not include zero) difference between c and c^’^ [53].

All analyses were conducted in SPSS version 26. We used Pearson’s *r* to examine correlations between the four variables (ASD diagnosis, CBCL internalizing, CBCL externalizing, and PQoL). The autism variable was dummy coded (0 for CG, 1 for autism) for the analyses. To test the conceptual model (Fig. 1), we used the SPSS macro, PROCESS version 3.4.1 [53], to test the serial indirect effects of internalizing and externalizing problems between autism and QoL. Mediation effects were estimated using 5000 bootstrapped samples and considered statistically significant if 95% confidence intervals (CI) did not include zero. Missing data were handled by listwise deletion. A serial mediation analysis isolates each mediator’s indirect effect and the indirect effect of both mediators together. As the mediators could act in the opposite order to the conceptual model, we tested this in separate models. Following up on the results from this analysis, we used simple mediation analysis to test the mediating effects of the three subscales on the scale of the internalizing problem: withdrawn-depressed, anxious-depressed, and somatic complaints. The mediating effects of these three variables were tested in the same analysis to control for the effect of the other variables.

## Results

### Correlations between variables

As expected, all four variables showed significant (two-tailed) correlations with each other (see Table 3). Autism was moderately related to reduced QoL and strongly related to more internalizing and externalizing problems. More internalizing and externalizing problems were moderately related to reduced QoL.

**Table 3:** Correlations between variables

	Autism^1^	Internalizing	Externalizing
Internalizing	0.70**		
Externalizing	0.59*	0.70**	
Quality of Life	− 0.34**	− 0.43**	− 0.30*

Note. *p < .05, ** p < .01; 1Point-biserrial correlations

### Serial mediation analysis

**Fig. 2:**
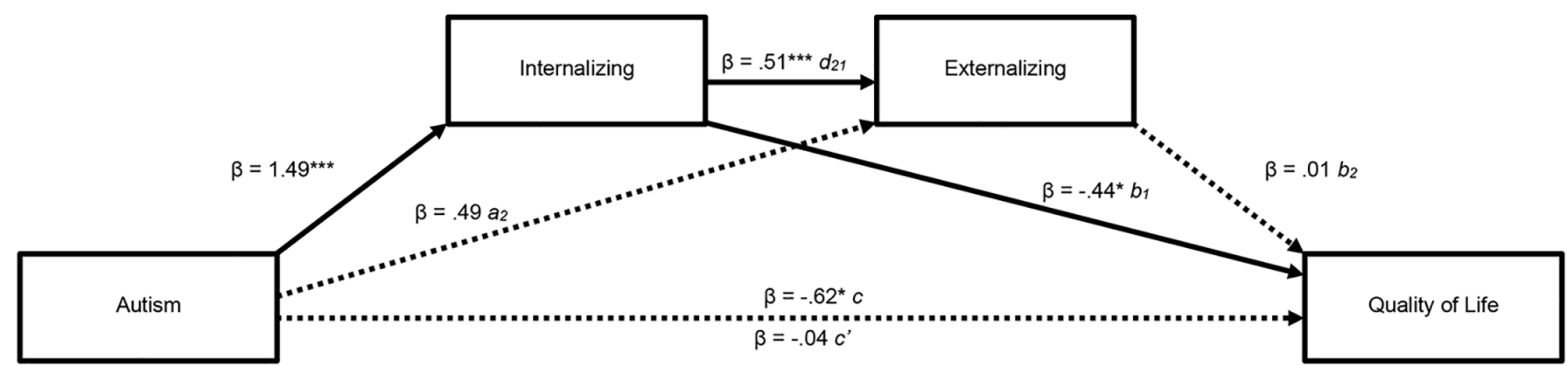
Serial multiple mediation of autism relation to Quality of Life where internalizing problems is entered as the first mediator into the model and externalizing problems next. ***p < .001. The values shown are standardized coefficients

There was a significant mediating effect of internalizing problems between autism and quality of life (mediating effect (a_1_ + b_1_): β = − 0.65, 95% CI [-1.22, − 0.04]). In contrast to our hypothesis, there was no significant mediating effect of externalizing problems on quality of life (mediating effect (a_2_ + b_2_): β = 0.01, 95% CI [-0.17, 0.23]) nor a serial mediating effect (mediating effect (a_1_, d_21_, b_2_): β = 0.01, 95% CI [-0.25, 0.23]). The total model accounted for 19% of the variance in quality of life (*F* [3, 59] = 4.51, *p* = .007, *R*^*2*^ = 0.186).

**Fig. 3:**
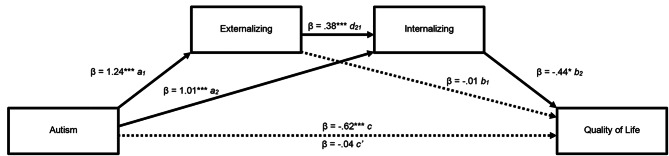
Serial multiple mediation of autism relation to Quality of Life where externalizing problems is entered as the first mediator into the model and internalizing problems next. ***p < .001. Values shown are standardized coefficients

Repeating the same analysis but switching places on internalizing and externalizing problems, supported the aforementioned results (see Fig. 3). There was no significant mediation effect of externalizing problems, but the mediation effect of internalizing problems remained significant (mediating effect (a_2_ + b_2_): β = − 0.45, 95% CI [-0.93, − 0.04]). However, in contrast to the previous analysis, the serial mediating effect was now significant (mediating effect (a_1_, d_21_, b_2_): β = − 0.21, 95% CI [-0.45, − 0.02]).

**Fig. 4:**
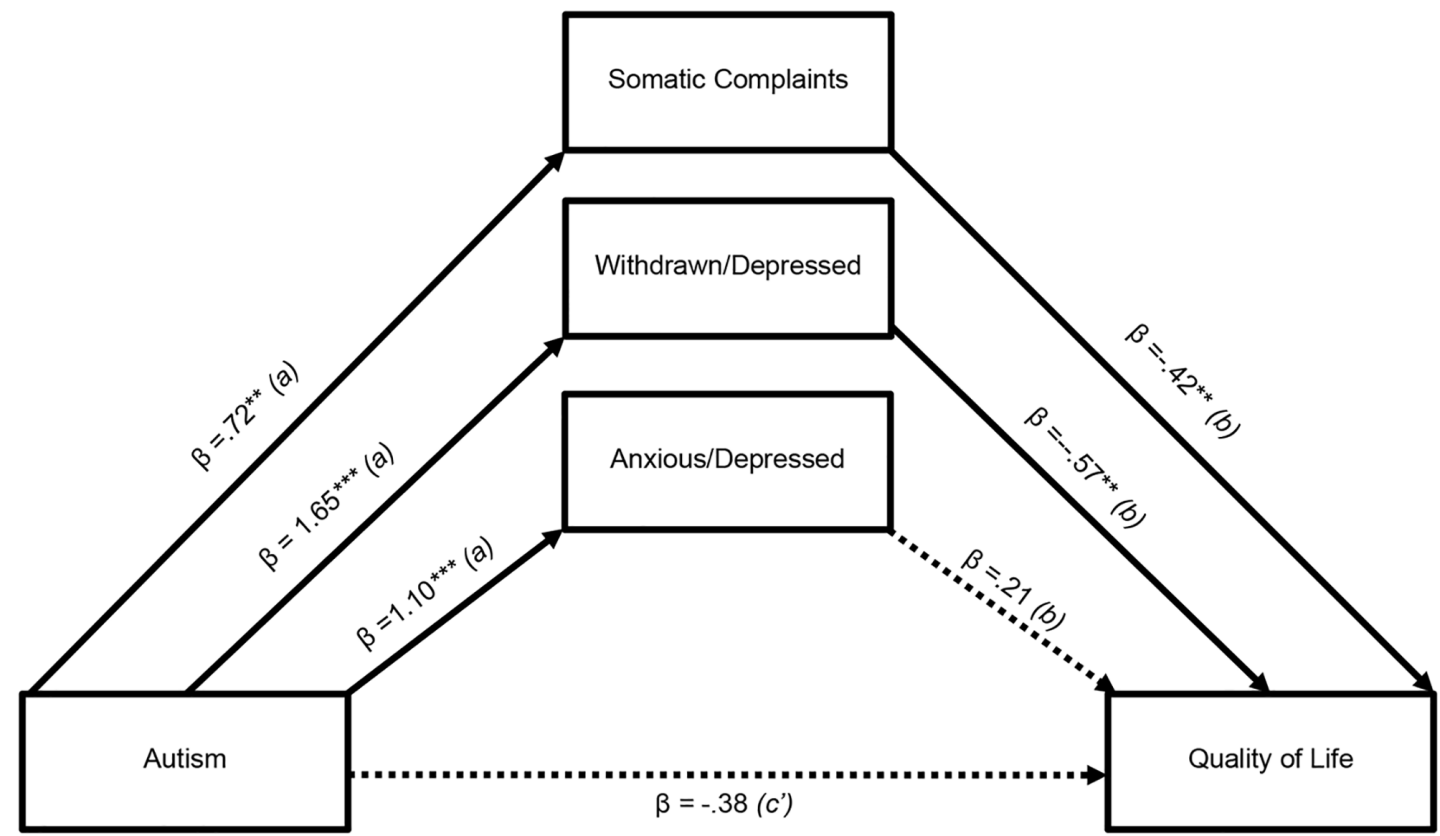
Parallel mediation of autism relation to Quality of Life where Anxious/Depressed, Withdrawn/Depressed and Somatic Complaints subscales of Adult Self-Report are entered simultaneously as mediators into the model ***p < .001. Values shown are standardized coefficients

Based on the two analyses, we concluded that internalizing problems seem to be the most prominent mediator between autism and reduced quality of life. We examined this mediating effect further by running a simple mediation analysis with the three subscales of the internalizing problems scale as parallel mediators (see Fig. 4). In this analysis, withdrawn-depressed (mediating effect (a + b): β = − 0.93, 95% CI [-1.85,-0.02]) and somatic complaints (mediating effect (a + b): β = − 0.30, 95% CI [-0.60,-0.03]) emerged as significant mediators.

## Discussion

First, we found that receiving a diagnosis of autism in childhood significantly predicted poorer PQoL in emerging adulthood. This is also in line with previous findings of the relationship between autism and subjective QoL [8, 13, 14, 16] and contrast to findings from two other studies reporting no relationship between autism and poor QoL [24] and [19]. One possible reason for divergent findings is the use of different measures when assessing QoL in people with autism. Some measures focus on health-related aspects of QoL while other aspects of life such as social inclusion or the possibility of using your full cognitive potential are underrepresented [54]. Our study expands on previous findings by demonstrating that autism is associated with reduced PQoL in a sample of emerging adults who were diagnosed with autism in childhood. This association might partly be exemplified in the qualitative study by Botha, Dibb [22] where the participants revealed great personal costs when trying to avoid the stigma of being labeled with autism in childhood and the unease they felt throughout adolescence into emerging adulthood as a result.

The hypothesis that internalizing problems in adolescence would mediate the relationship between receiving an autism diagnosis in childhood and PQoL in emerging adulthood was supported. This corroborates previous studies, which consistently have found a negative association between internalizing problems and QoL in adults with autism (see 26 for a review). However, unlike previous studies, we tested the mediation effect of internalizing problems on the relationship between an autism diagnosis and PQoL in a longitudinal design. The results from our analyses showed that when accounting for internalizing problems, autism diagnosis was no longer reliably associated with reduced PQoL. Internalizing problems fully mediated the relationship between an autism diagnosis and PQoL. This expands on the cross-sectional findings of Reed, Giles [55] demonstrating that social anxiety, depression, and loneliness fully mediated the relationship between autistic traits and subjective QoL in a group of university students. Our findings clearly emphasize the need to target internalizing problems among adolescents with autism. These findings are important as individuals with autism have been found to suffer from a higher burden of psychopathology symptoms across the lifespan than other clinical groups [56]. Previous findings from our sample have highlighted the persistence of internalizing problems from childhood into emerging adulthood among individuals with autism [36]. Adults with autism have claimed that interventions in childhood are important for improved adult QoL [57]. So, an important topic for future research is whether interventions targeting internalizing problems among children and adolescents with autism can contribute to better QoL in emerging adulthood.

Internalizing problems is critical to understanding QoL outcomes in adults with autism, but more research is needed to determine which factors influence internalizing problems at different stages of life. Better cognitive functioning in childhood seems to be associated with fewer problems with adult everyday functioning [58]. Among emerging adults with autism, Hollocks, Charman [59] found that deficits in cognitive flexibility contributed to internalizing problems. As a result, the core deficits in executive functions demonstrated by many adolescents with autism [38] may be relevant factors in understanding the elevated levels of internalizing problems and ultimately QoL. Furthermore, there may be specific factors influencing adolescent internalizing problems and QoL in the developmental phase of emerging adulthood. This period from adolescence and into emerging adulthood is a period of transition from living with parents to more independent living. This transition is often accompanied by a shift in school (e.g., from high school to college) or from school to work, and there is also a shift in services. Studies have highlighted a possible drastic decrease in services during this phase [7]. A large transition with increasing environmental demands, accompanied by a decrease in services and support, can contribute to more internalizing problems and poorer QoL in emerging adults with autism. Qualitative studies have revealed that emerging adults with autism frequently face a lack of personalized support and experience situations that are inadequate in meeting their psychosocial requirements [57, 60]. More research is needed within this domain to better understand, in-depth, the experiences of emerging adults with autism, and their needs for services and support.

In contrast to our hypothesis, the serial mediation showed no significant independent effect of externalizing problems on PQoL in either of the analyses. As there was a strong relationship between internalizing and externalizing problems, we expected both to act as mediators in succession, and that externalizing problems were affected by internalizing. Our analyses support the conceptual model in the way that internalizing problems fully mediated the relationship between autism and externalizing problems, while there was only partial mediation in the opposite direction, where autism still had a significant relationship with internalizing problems after entering externalizing problems as a mediator. However, there was no significant relation between externalizing problems and PQoL, and thus, our serial mediation model was not supported. One explanation for this may be that the level of externalizing problems was not very high in our sample. Externalizing problems T-scores for the autism group were closer to the population mean and the CG mean than internalizing problems scores, and they were less correlated with the autism diagnosis than internalizing problems were.

The broadband scale of internalizing problems on the Child Behavior Checklist consists of three related, but distinct, subscales: anxious/depressed, withdrawn/depressed, and somatic complaints. As internalizing problems were found to have such a large impact on the relationship between autism and PQoL, we wanted to investigate how these three sub-scales acted in parallel mediation analysis. We found a significant relationship between autism and all three subscales. However, the anxious/depressed subscale did not reach significance as a mediator between autism and PQoL. This could be due to a higher level of symptom overlap between withdrawn/depressed and autism than between anxious/depressed and autism. Depression has been identified as a major contributor to reduced subjective QoL in people with autism [28, 29]. Further, levels of depression in emerging adults with autism have been reported to be of similar magnitude to that of emerging adults with a primary depressive disorder [28]. We have previously found a significant positive correlation between changes in autism symptoms and changes in depressive symptoms in our sample over two years [61]. This indicated that having autism and the possible stigma associated with this may lead to chronic conflicts, misunderstandings, and failures in professional life [62]. As such, it is possible that ameliorating diagnosis-related stigma and depressive symptoms may not only lead to improved subjective QoL but also reduce perceived stress related to autism.

Somatic complaints are common among children experiencing trauma and are associated with anxiety [63]. Ongoing potentially traumatizing school bullying or other adverse childhood experiences related to having a diagnosis of autism are relatively common among adolescents with autism and these are also more likely to present themselves as somatic complaints [64]. It is a possibility that the somatic complaints reported here could be prodromal to later anxiety problems common in autism. The mediating effect of adolescent somatic complaints on later subjective QoL may be caused by somatic complaints in adolescent age developing into anxiety which hamper subjective QoL in emerging adulthood.

Another point to be made is that the autism diagnosis was informed by clinical interview and based on clinical decisions. Thus, by using the diagnosis rather than parent-reported symptoms, our mediation model utilizes cross-informant effects (clinician-decided diagnosis, parent-reported internalizing, and externalizing problems, self-reported QoL), and avoids the issue of common method bias, which usually inflates correlations and contributes to higher explained variance. As such, our use of cross-informant effects may partially explain the moderate amount of variance explained by the model.

### Implications and future directions

This study emphasizes the importance of assessing and treating mental health issues such as depression when seeking to improve the quality of life for emerging adults with autism. For the general public, symptoms of autism may be seen as the main driver of QoL for individuals with autism. Our study reveals that this is not necessarily the case. Mental health issues should therefore be considered as a comorbid trait with great importance for QoL and not as an inevitable part of having autism. This notion is following the findings of Joshi, Wozniak [56]. Furthermore, they underline the importance of autism-specific interventions when treating comorbid psychopathology. However, there is no general agreement upon what constitutes a good QoL for people with autism [13]. QoL may be perceived as something different for people with autism than for people in the typical population. Autism is commonly regarded as a disadvantage affecting QoL negatively, partly because of the stigma associated. It is therefore important for future research to focus on stigma management both on an individual as well as on a societal level [22]. Depending on the context, autistic characteristics can also be regarded by adults with autism as a strength [65, 66] and this strength perspective might help reframe common misconceptions about autism. It is important in the future to gain knowledge on how quantitatively measured difficulties affect perceived QoL and which factors constitute a good QoL for emerging adults with autism [67].

### Limitations

One obvious limitation of this study is the relatively small number of participants and the complete case analysis. Conducting the analyses only on participants with complete data on all measurement occasions may introduce some bias to the estimates. However, given that PQoL was only measured on one occasion, the data was not suitable for multiple imputation, and other forms of imputation (e.g., mean imputation) may have introduced an even larger bias to the estimates. Despite the limitation of a relatively small number of participants and a complete case analysis, a notable strength is a relatively high retention rate over 10 years (76% retention). Furthermore, there was no significant differences between those retained and those not-retained at the 10-year follow-up, supporting the validity of the estimates. Traditional measures of QoL such as the PQoL may not sufficiently tap into what QoL is for people with autism [60], and we did not measure stigma and the possible stigmatizing effects of having an autism diagnosis. The results from this study may not be generalized to less cognitively able people with autism (IQ < 70). Another limitation may be that our participants are drawn from a clinical population that has or has had greater psychosocial problems in their childhood and that we have no measure of a possible perceived burden of having autism. Still, we believe that the longitudinal design adds important knowledge on the relationship between having been diagnosed with autism in childhood and QoL in emerging adulthood.

## Conclusion

Taken together, our findings indicate that reduced quality of life in emerging adults with autism is mediated by internalizing problems in adolescence, rather than growing up with autism per se.

Interventions aimed at improving co-existing symptoms of depression and somatic complaints in adolescence may be a more effective target for improved current and later QoL than interventions aimed at autism.

## Data Availability

The data that support the findings of this study are available from PA, but restrictions apply to the availability of these data, which were used under license for the current study, and so are not publicly available. Data are however available from the authors upon reasonable request and with permission of ES (PI).

## Footnotes

1 In accordance with the practice of the Norwegian Autism Society this article uses person-first language.

## List of abbreviations

ADHD: attention -deficit/hyperactivity disorder
AQ: autism quotient scale
AQ 10: autism quotient scale 10 questions version
ASD: autism spectrum disorder
ASDI: Asperger syndrome diagnostic interview
ASSQ: autism spectrum screening questionnaire
CBCL: child behavior checklist
CG: comparison group
CI: confidence interval
K-SADS-PL: Schedule for Affective Disorders and Schizophrenia for School-Aged Children/Present and Lifetime version
LINEUP: Lillehammer neurodevelopmental follow-up study
PQoL: perceived quality of life
QoL: quality of life
TS: Tourette’s syndrome
WASI: Wechsler abbreviated scale of intelligence
